# Risk Factors and Clinical Impacts of Post-Pancreatectomy Acute Pancreatitis After Pancreaticoduodenectomy: A Single-Center Retrospective Analysis of 298 Patients Based on the ISGPS Definition and Grading System

**DOI:** 10.3389/fsurg.2022.916486

**Published:** 2022-07-04

**Authors:** Shuai Wu, Hanxue Wu, Guiping Xu, Yaling Zhao, Feng Xue, Shunbin Dong, Liang Han, Zheng Wang, Zheng Wu

**Affiliations:** ^1^ Department of Hepatobiliary Surgery, the First Affiliated Hospital of Xi’an Jiaotong University, Xi’an, China; ^2^ Department of Physiology and Pathophysiology, School of Basic Medicine, Xi’an Jiaotong University, Xi’an, China; ^3^ Department of Radiology, the First Affiliated Hospital of Xi’an Jiaotong University, Xi’an, China; ^4^ Department of Epidemiology and Biostatistics, School of Public Health, Xi’an Jiaotong University Health Science Center, Xi’an, China

**Keywords:** pancreaticoduodenectomy, acute pancreatitis, postoperative complications, risk factors, retrospective analysis

## Abstract

**Background:**

The definition and grading system of post-pancreatectomy acute pancreatitis (PPAP) has recently been proposed by ISGPS. This study aimed to put this definition and classification into practice and investigate the potential risk factors and clinical impacts of PPAP.

**Methods:**

Demographic and perioperative data of consecutive patients who underwent pancreaticoduodenectomy (PD) from January 2019 to July 2021 were collected and analyzed retrospectively. The diagnostic criteria of PPAP published by ISGPS, consisting of biochemical, radiologic, and clinical parameters, were adopted. The risk factors were analyzed by univariate and multivariate analyses.

**Results:**

A total of 298 patients were enrolled in this study, and the total incidence of PPAP was 52.4% (150 patients). Stratified by clinical impacts of PPAP, the incidences of grades B and C PPAP were 48.9% and 3.5%, respectively. PPAP after PD was significantly associated with pancreatic fistula and other unfavorable complications. Soft pancreatic texture (OR 3.0) and CRP ≥ 180 mg/L (OR 3.6) were the independent predictors of PPAP, AUC 0.613. Stratified by the grade of PPAP, soft pancreatic texture (OR 2.7) and CRP ≥ 180 mg/L (OR 3.4) were the independent predictors of grade B PPAP, and soft pancreatic texture (OR 19.3), operation duration >360 min (OR 13.8), and the pancreatic anastomosis by using conventional duct to mucosa methods (OR 10.4) were the independent predictors of grade C PPAP. PPAP complicated with pancreatic fistula significantly increased the severe complications and mortality compared to only PPAP occurrence.

**Conclusion:**

PPAP was not an uncommon complication after PD and was associated with unfavorable clinical outcomes, especially since it was complicated with pancreatic fistula. Soft pancreatic texture and CRP ≥ 180 mg/L were the independent predictors of PPAP. Higher-volume multicenter and prospective studies are strongly needed.

## Introduction

Post-ERCP pancreatitis (PEP) has been widely recognized, and its clinical practice guidelines have been published (1). Under the same postoperative background, postpancreatectomy acute pancreatitis (PPAP) was not comprehensively recognized. Previous studies regarded PPAP as an indirect manifestation of pancreatic fistula (PF) (2, 3). PPAP has attracted attention since Connor proposed the first definition based on the systematic review (4). Several medical centers carried out their clinical studies relevant to PPAP (5–9), and the incidence reported in previous studies varied widely from 1.5% to 67.9% due to the lack of authoritative definitions and terminology.

Recently, the international study group of pancreatic surgeons (ISGPS) developed a consensus definition, diagnostic, and grading criterion of PPAP. PPAP is defined as acute inflammation of the remnant pancreas within the first 3 days after partial pancreatectomy. The ISGPS group come up with the term “PPAP” instead of postoperative pancreatitis (POAP) (4) to refer specifically to pancreatitis after partial pancreatectomy. This group also clarified the definition of postoperative serum hyperamylasemia (POH), which had previously been confused with PPAP (10). The diagnostic criteria of PPAP (11) require three dimensions: sustained POH, clinical impacts relevant to PPAP, and radiologic features of acute pancreatitis (12, 13). The grading system of PPAP is based on clinical impacts, including POH (biochemical change only), grade B (mild or moderate clinical impacts), and grade C (severe clinical impacts).

Here, in this study, we used the definition and grading system of PPAP that had just been published by ISGPS to review our clinical data and aimed to assess PPAP in our clinical practice and recognize potential risk factors of PPAP.

## Methods

### Patients and Data Collection

This retrospective study was performed on all patients who consecutively underwent pancreaticoduodenectomy (PD) from January 2019 to July 2021 at the First affiliated Hospital of Xi’an Jiaotong University. Patients who got a PD procedure in the Department of Hepatobiliary Surgery were enrolled in this study. Patients without a detailed record of postoperative complications, serum amylase, and abdominal CT scan in the early postoperative period were excluded. This study was approved by the local ethics committee (ethical approval number: XJTU1AF2015LSL-057), and informed consent was obtained from the patients.

To control bias, demographics, preoperative clinical parameters, and postoperative clinical parameters were collected by different individuals to reduce the behavior of artificial adjustment and the influence of personal tendency in the data collection phase. Demographic characteristics included age, gender, body mass index (BMI), smoking, and drinking conditions. Past medical history and comorbidities included cardiovascular and pulmonary diseases, hepatitis, kidney diseases, cholelithiasis, type 2 diabetes mellitus, history of acute pancreatitis attack and previous abdominal surgery, preoperative jaundice, and the American Society of Anaesthesiologists (ASA) score.

Operative details included blood loss, transfusion, and operative duration. The surgical procedure details also contained whether pylorus-preserving pancreaticoduodenectomy (PPPD) (14) or Whipple procedure (15), standard or extended resection (vascular resection and/or extended organ resection), the usage of pancreatic duct stent, and the diameter of the pancreatic duct. The pancreatic texture was assessed by the primary surgeon and documented in the surgical records. The fistula risk scores (16) were calculated. The management of pancreatic stump in operations was all treated with pancreatojejunostomy (PJ), none of the pancreatogastric anastomosis (PG), including modified Blumgart pancreatic duct–mucous anastomosis, double-layer duct-to-mucosa (conventional duct to the mucosa), and end-to-side or end-to-end invagination. Pathology types were divided into three groups: the first was pancreatic ductal adenocarcinoma (PDAC) and chronic pancreatitis (CP), the second was another malignant group (periampullary carcinoma, duodenal carcinoma), and the third was the benign and low malignant group (pancreatic cyst tumor, pancreatic neuroendocrine tumor, duodenal stromal tumor, and other benign or precancerous lesions).

The removal time and volume of drainage tubes, including gastric, urine, and abdominal drainage tubes, were collected by the medical orders and medical records. The number of patients who accepted the neoadjuvant therapy was collected. The usage of the pancreatic exocrine inhibitory drug (octreotide or somatostatin) and ulinastatin was recorded. The length of hospital stay, postoperative hospital stay, intensive care unit (ICU) stay, postoperative mortality, and gross cost were collected. Perioperative serum biochemical markers including CRP (on POD 0–3), total bilirubin (TB), direct bilirubin, albumin (ALB), calcium, and amylase were also recorded.

### Definitions

The preoperative jaundice state means serum TB ≥ 34.2 μmol/L (more than 2 times the upper normal serum level) before the surgery. The liquid intake/output volume was defined as the difference between all intake volume and output (urine volume plus blood loss) on the day of operation (POD 0) including intraoperative. About 2000 ml (roughly the physiological requirements) was the cutoff to assess the liquid intake/output volume. The measures of preoperative biliary drainage include percutaneous transhepatic biliary drainage (PTBD), endoscopic nasobiliary drainage (ENBD), and T-shaped tube placed in the previous operation. The volume of abdominal drainage, namely, extraintestinal drainage on the postoperative day, did not include the intestinal drainage such as the pancreatic duct stents, biliary stents, PTBD, and gastric tubes. Hypoalbuminemia was defined as the serum concentration on POD 1 of less than 3.5 g/dL (35 g/L), and serum calcium on POD 1 below the lower limit was defined as hypocalcemia. The ΔTB was equal to the postoperative minus preoperative TB value on POD 1. The unchanged ΔTB ranged from −5 μmol/ L to 5μmol/ L, higher than that defined as elevation and lower than that defined as decrease.

The definition and severity of PPAP (11), PF (17), delayed gastric empty (DGE) (18), and postpancreatectomy hemorrhage (PPH) (19) were according to ISGPS. Bile leakage (BL) was defined as the concentration of bilirubin in the drainage fluid >3 times of the serum bilirubin on or POD 3 or requiring radiological or surgical intervention due to biliary collection or biliary peritonitis (20). Intra-abdominal infection was supported by evidence of bacterial culture etiology in the abdominal drainage fluid. Wound infection was proved by purulent discharge or the need to remove the suture and drainage. Acute kidney injury (AKI) was according to the Kidney Disease Improving Global Outcomes (KDIGO) classification (21). The abdominal fluid collection was confirmed by imaging (ultrasound or CT scans). Percutaneous drainage was guided by ultrasound or CT scans under local infiltration anesthesia. Unplanned reoperation meant the unplanned need for laparotomy or interventional surgery during the hospital stay. The severity of complications was according to the Clavien–Dindo classification (22); the ≥IIIb complications were defined as serious complications. Postoperative mortality was stipulated as mortality within 30 days after surgery.

### Evaluation of Postoperative CT

**Figure 1:**
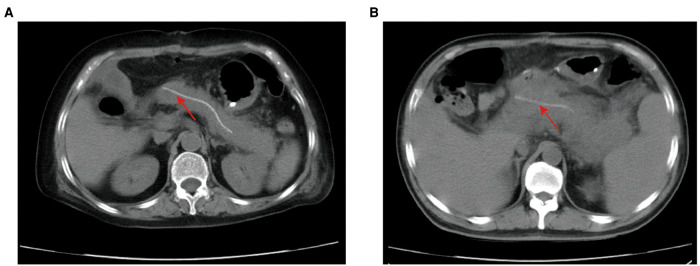
Postoperative CT scans of PPAP. (**A**) Acute edematous pancreatitis after PD: the boundary of the remnant pancreas is coarse, extensive exudation and inflammatory change around the remnant pancreas, not the surgical field. (**B**). Acute necrotizing pancreatitis after PD: the borderline of remnant pancreas is not distinct, inflammatory changes and exudate surrounding the remnant pancreas, necrosis change in the pancreatic parenchyma and the peripancreatic tissue. Red arrow: internal pancreatic duct stent. PPAP, postoperative acute pancreatitis; PD, pancreaticoduodenectomy.

Postoperative CT scans were evaluated by the team of the Department of Radiology at the First Affiliated Hospital of Xi’an Jiaotong University, which consisted of one professor and two associate professors majoring in the abdominal area. This team reached a consensus on the manifestations of PPAP on postoperative CT images (Figure 1). Based on the radiological features in the early postoperative period (11, 12), PPAP can be stratified into acute edematous pancreatitis and acute necrotizing pancreatitis. Interstitial edematous pancreatitis shows relatively homogeneous enhancement or attenuation, inflammatory change, and peripancreatic fluid collection (Figure 1A), while acute necrotizing pancreatitis shows inhomogeneous enhancement or attenuation, necrosis of the pancreatic parenchyma and/or the peripancreatic tissue (Figure 1B).

### Statistics

SPSS 21.0 software package was used for data processing. The normal distribution data are described by $\bar{x}\pm s$, the non-normal distribution data are described by median (IQR), and the counting data are described by proportion, relative ratio, and composition ratio. The Mann–Whitney *U* test for two independent samples was used for non-normal distribution. Pearson’s chi-square test was used for counting data, and the *t*-test was used for two independent samples in accordance with normal distribution. Univariate analysis was used to judge the association between perioperative parameters and PPAP. Multivariate analyses, including binary and Firth logistic regression, were used to recognize the risk factors of PPAP and PF. The efficiency of the predicting model was measured by ROC curve analysis. *P* values <0.05 were defined as statistically significant.

## Results

### Patients’ Characteristics

**Figure 2:**
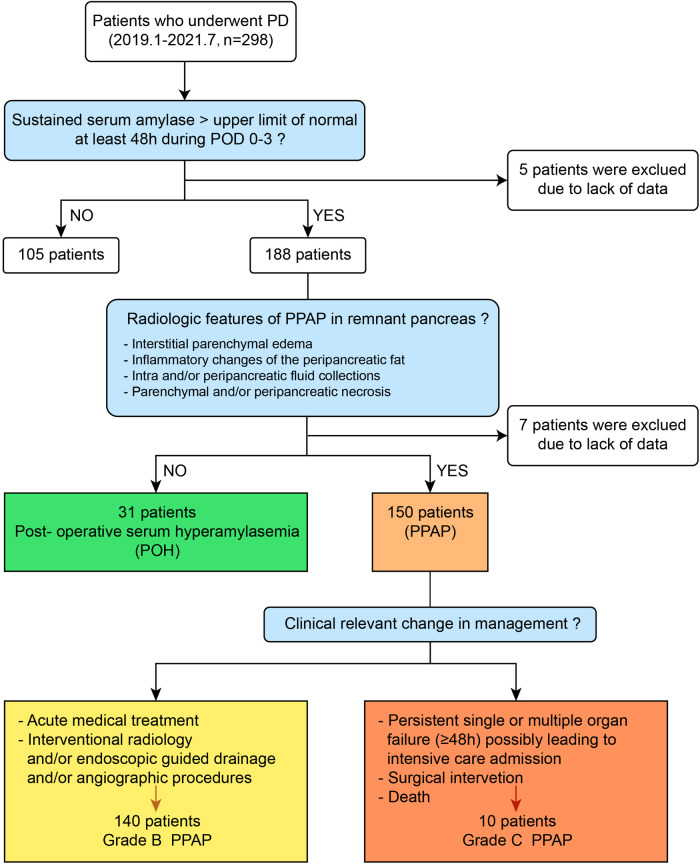
Diagnostic flow chart of PPAP based on the diagnostic criteria of ISGPS.

A total of 298 consecutive patients who underwent PD from January 2019 to July 2021 at the First affiliated Hospital of Xi’an Jiaotong University were enrolled in this study. Twelve (4.0%) patients were excluded due to the lack of detailed records of postoperative complications, serum amylase, and abdominal CT scan data. The sex ratio of men to women was 1.6:1. The mean age of the patients was 62 (55–69) years. Twenty-three (8%) patients had a history of acute pancreatitis attack, and 71 (24.8%) had cholelithiasis. Sixty-two (21.7%) patients had abdominal surgery previously. The most frequent indications for PD were malignant tumors, including 92 (32.2%) pancreatic ductal adenocarcinoma, 157 (54.9%) periampullary carcinoma, and 6 (2.1%) duodenal carcinoma. The residual contains 16 (5.6%) pancreatic cyst tumor, 4 (1.4%) pancreatic neuroendocrine tumor, 3 (1.0%) chronic pancreatitis, 2 (0.7%) duodenal stromal tumor, and 6 (2.1%) other benign or precancerous lesions. According to diagnostic criteria of PPAP formulated by ISGPS, the patients were divided into the PPAP group and non-PPAP group (the diagnostic flow chart is shown in Figure 2); the total incidence of PPAP was 52.4% (150 patients). Stratified by clinical impacts of PPAP, grade B PPAP was 48.9% (140 patients) and grade C PPAP was 3.5% (10 patients). The serum amylase level on POD 1–3 is shown in Supplementary Table 1 (the normal upper limit of serum amylase in our institution is 135 U/L). The incidence of clinically relevant pancreatic fistula (CR-PF) was 23.4% (67 patients). Of the patients with CR-PF, 56 (19.6%) patients had grade B PF and 11 (3.8%) patients had grade C PF.

### PPAP After PD Was Associated with Unfavorable Complications

The postoperative outcomes of patients grouped by the occurrence of PPAP are shown in Table 1. The complications of Clavien–Dindo ≥ IIIb were significantly increased in patients with PPAP, and CR-PF, intra-abdominal infection, abdominal fluid collection, puncture, and drainage treatment, and mortality were also significantly increased. The total lengths of hospital stay and postoperative hospital stay were longer in the PPAP group. The hospitalization cost in the PPAP group was also significantly increased by $1,721 ($15,671 and $13,950, respectively).

**Table 1:** Postoperative outcomes stratified by the occurrence of PPAP.

	PPAP		*Z/χ^2^*	*p*-value
	No (*n* = 136)	Yes (*n* = 150)		
Clavien–Dindo, *n* (%)			5.637	0.018
<IIIb	133 (97.8)	137 (91.3)		
≥IIIb	3 (2.2)	13 (8.7)		
CR-PF, *n* (%)			18.942	<0.001
B	15 (11.0)	41 (27.3)		
C	2 (1.5)	9 (6.0)		
PPH, *n* (%)			6.016	0.111
A	9 (6.6)	11 (7.3)		
B	2 (1.5)	8 (5.3)		
C	5 (3.7)	12 (8.0)		
DGE, *n* (%)			4.203	0.240
A	34 (25.0)	28 (18.7)		
B	20 (14.7)	32 (21.3)		
C	6 (4.4)	11 (7.3)		
BL, *n* (%)	3 (2.2)	1 (0.7)	0.363	0.547
Intra-abdominal infection, *n* (%)	52 (38.2)	78 (52.0)	5.451	0.020
AKI, *n* (%)	2 (1.5)	2 (1.3)	0.010	0.921
Bowel obstruction, *n* (%)	4 (2.9)	4 (2.7)	0.020	0.888
Abdominal fluid collection, *n* (%)	28 (20.6)	71 (47.3)	22.543	<0.001
percutaneous drainage, *n* (%)	9 (6.6)	28 (18.7)	9.194	0.002
Secondary operation, *n* (%)	4 (2.9)	12 (8.0)	3.456	0.063
Mortality, *n* (%)	0 (0)	6 (2.1)	5.557	0.018
ICU stay (day)	1.0 (0–2.0)	1.0 (0–2.0)	−0.332	0.740
Total hospital stay (day)	23.0 (18.0–28.0)	24.0 (20.0–31.0)	−2.000	0.045
Postoperative hospital stay (day)	15.5 (12.0–19.0)	17.0 (14.0–21.3)	−2.520	0.012
Cost ($)	13,950 (11,601–17,041)	15,671 (12,925–19,124)	−2.778	0.005

*PPAP, postpancreatectomy acute pancreatitis; CR-PF, clinical relevant pancreatic fistula; PPH, postpancreatectomy hemorrhage; DGE, delayed gastric empty; BL, bile leakage; AKI, acute kidney injury; ICU, intensive care unit.*

To further evaluate the effects of PPAP on serious postoperative complications, univariate and multivariate analyses were conducted on patients with Clavien–Dindo ≥ IIIb (Table 2). This indicated that PPH (OR 12.807, 95% CI 2.401–131.105), intra-abdominal infection (OR 11.101, 95% CI 1.158–1617.774), AKI (OR 97.612, 95% CI 2.942–34098.204), and postoperative hypoalbuminemia (OR 4.166, 95% CI 1.063–19.866) were independent predictors of Clavien–Dindo ≥ IIIb. It' is worth noting that in univariate analyses, there was statistical difference in PPAP, suggesting that PPAP did not increase the incidence of Clavien–Dindo ≥ IIIb directly.

**Table 2:** Univariate and multivariate analyses of Clavien–Dindo  ≥ IIIb postoperative complications.

	Univariate			Multivariate		
	No (*n* = 270)	Yes (*n* = 16)	*p*-value	OR	95% CI	*p*-value
PPAP, *n* (%)	137(50.7)	13(81.3)	0.018	2.464	0.358–13.477	0.268
CR-PF, *n* (%)	56 (20.7)	11 (68.8)	<0.001	1.642	0.107–3.043	0.545
PPH, *n* (%)	34 (12.6)	13 (81.3)	<0.001	12.807	2.401–131.105	0.002
DGE, *n* (%)	115 (42.6)	16 (100)	<0.001	6.373	0.592–759.752	0.137
BL, *n* (%)	3 (1.1)	1 (6.3)	0.207			
Intra-abdominal infection, *n* (%)	114 (42.2)	16 (100)	<0.001	11.101	1.158–1617.774	0.034
Abdominal fluid collection, *n* (%)	86 (31.9)	13 (81.3)	<0.001	1.925	0.358–13.477	0.445
AKI, *n* (%)	1 (0.4)	3 (18.8)	0.001	97.612	2.942–34,098.204	0.005
Bowel obstruction, *n* (%)	8 (3.0)	0 (0)	1			
Wound infection, *n* (%)	2 (0.7)	1 (6.3)	0.159	24.047	0.085–10,249.661	0.608
Hypoalbuminemia, *n* (%)	96 (35.6)	13 (81.3)	<0.001	4.166	1.063–19.866	0.041
Hypocalcemia, *n* (%)	169 (62.6)	11 (68.8)	0.620			

*PPAP, postpancreatectomy acute pancreatitis; CR-PF, clinically relevant pancreatic fistula; PPH, post-pancreatectomy hemorrhage; DGE, delayed gastric empty; BL, bile leakage; AKI, acute kidney injury.*

### Risk Factors for PPAP After PD

**Figure 3:**
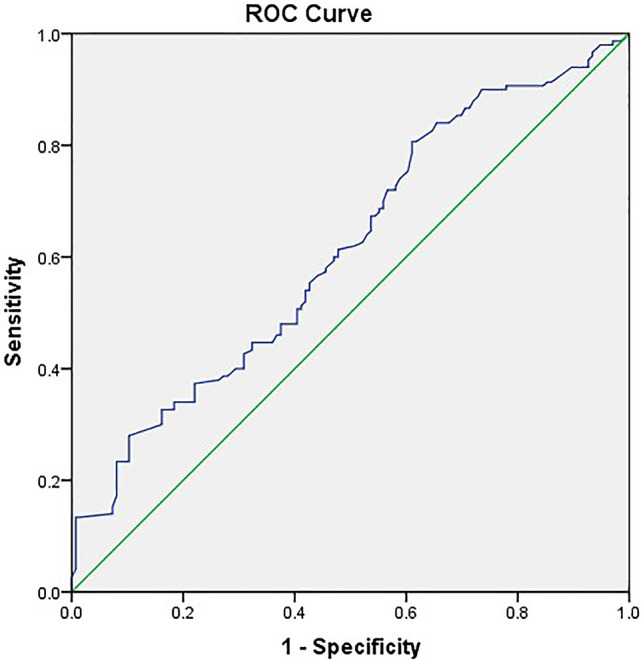
Receiver operating characteristic (ROC) curve for threshold analysis of predicting PPAP (AUC 0.613).

Stratified by the PPAP occurrence, the perioperative characteristics as well as univariate and multivariate analyses are shown in Table 3. Female, BMI ≥ 25, preoperative jaundice state, pancreatic texture, diameter of pancreatic duct, the techniques of pancreatic anastomosis, and CRP ≥ 180 mg/L were enrolled into the multivariate analysis model. Due to multicollinearity of the diameter of pancreatic duct and pancreatic texture, fistula risk scores were excluded from multivariate analysis. The soft texture of pancreatic stump and CRP ≥ 180 mg/L were defined as independent predictors of PPAP through multivariate analysis (OR 2.953, 95% CI 1.764–4.943 and OR 3.591, 95% CI 2.047–6.297, respectively). The area under the ROC curve was 0.613 (Figure 3).

**Table 3:** Univariate and multivariate analyses of PPAP.

	Univariate analysis			Multivariate analysis		
	No (*n* = 136)	Yes (*n* = 150)	*p*-value	OR	95% CI	*p*-value
Sex ratio (M:F)	95:41	81:69	0.006*	1.560	0.871–2.794	0.135
Age (year)	62 (55–69)	62 (56–69)	0.786			
BMI ≥ 25, *n* (%)	81 (59.6)	74 (49.3)	0.083	1.389	0.788–2.448	0.256
Tobacco use, *n* (%)	44 (32.4)	40 (26.7)	0.292			
Alcohol use, *n* (%)	14 (10.3)	13 (8.7)	0.638			
AP history, *n* (%)	10 (7.4)	13 (8.7)	0.683			
DM, *n* (%)	20 (14.7)	18 (12.0)	0.501			
Hypertension, *n* (%)	35 (25.7)	40 (26.7)	0.858			
Cholelithiasis, *n* (%)	32 (23.5)	39 (26.0)	0.629			
Preoperative jaundice state, *n* (%)	88 (64.7)	81 (54.0)	0.066	1.292	0.750–2.223	0.356
Previous abdominal surgery, *n* (%)	30 (22.1)	32 (21.3)	0.882			
ASA, *n* (%)			0.260			
1	1 (0.7)	4 (2.7)				
2	102 (75.0)	102 (68.0)				
≥3	33 (24.3)	44 (29.3)				
Vascular resection, *n* (%)	15 (11.0)	18 (12.0)	0.798			
Extended organ resection, *n* (%)	2 (1.5)	4 (2.7)	0.770			
Pancreatic duct diameter <5 mm, *n* (%)	74 (54.4)	105 (70.0)	0.007	1.534	0.883–2.663	0.129
Pancreatic texture, *n* (%)			<0.001	2.966	1.733–5.076	<0.001
Soft	46(33.8)	96(64.0)				
Hard	90(66.2)	54(36.0)				
Pancreatic stent, *n* (%)			0.395			
Unused	14(10.3)	9(6.0)				
Internal	89(65.4)	105(70.0)				
External	33(24.3)	36(24.0)				
Bleeding ≥ 400 ml, *n* (%)	80 (58.8)	77 (51.3)	0.204			
liquid intake/output volume ≤ 2000 ml, *n* (%)	34 (25.0)	39 (26.0)	0.846			
Operation duration (min)	360 (300–420)	360 (296–434)	0.291			
Fistula risk scores, *n* (%)			0.017	/		
Negligible	17 (12.5)	13 (8.7)		/		
Low	50 (36.8)	34 (22.7)		/		
Moderate	63 (46.3)	97 (64.7)		/		
High	6 (4.4)	6 (4.0)		/		
Pathology type, *n* (%)			0.386			
PDAC and CP	50 (36.8)	45 (30.0)				
Other malignant	75 (55.1)	88 (58.7)				
Benign and low malignant	11 (8.1)	17 (11.3)				
Pancreatic anastomosis, *n* (%)			<0.001			
Modified Blumgart	85 (62.5)	63 (42.0)		0.767	0.304–1.934	0.574
Conventional duct to mucosa	36 (26.5)	75 (50.0)		1.854	0.712–4.829	0.206
Invagination	15 (11.0)	12 (8.0)		1		
Pancreatic enzymes inhibitors, *n* (%)			0.086			
Unused	3 (2.2)	0				
Octreotide	63 (46.3)	60 (40.0)				
Somatostatin	70 (51.5)	90 (60.0)				
Ulinastatin use	39 (28.7)	50 (33.3)	0.396			
Hypoalbuminemia, *n* (%)	54 (39.7)	55 (36.7)	0.597			
CRP ≥ 180 mg/l, *n* (%)	30 (22.1)	77 (51.3)	<0.001	3.591	2.047–6.297	<0.001
Hypocalcemia, *n* (%)	88 (64.7)	92 (61.3)	0.555			
ΔTB, *n* (%)			0.623			
Decrease	61 (44.9)	59 (39.3%)				
Unchanged	21 (15.4%)	27 (18.0%)				
Elevation	54 (39.7%)	64 (42.7%)				
POD 1 drainage volume (ml)	67.5 (31.3–157.5)	80.0 (32.5–215.0)	0.535			
POD 2 drainage volume (ml)	72.5 (30.0–160.0)	90.0 (25.0–200.0)	0.748			
POD 3 drainage volume (ml)	81.0 (20.8–178.8)	80.0 (20.0–182.5)	0.865			

*PPAP, postpancreatectomy acute pancreatitis; AP, acute pancreatitis; DM, diabetes mellitus; ASA, American Society of Anesthesiologists; PDAC, pancreatic duct adenocarcinoma; CP, chronic pancreatitis; ΔTB, postoperative minus preoperative total bilirubin value; POD, postoperative days.*

In this study, 10 patients occurred gade C PPAP in the patients’ cohort, and the incidence of grade C PPAP was 3.5%. Grade C PPAP was a rare but life-threatening complication after PD. Stratified by the grade of PPAP, the perioperative characteristics as well as univariate analysis are shown in Table 4. Compared to the non-PPAP group, male, BMI ≥ 25, preoperative jaundice state, pancreatic duct diameter <5 mm, soft pancreatic texture, the methods of pancreatic anastomosis, and CRP ≥ 180 mg/L were enrolled into the multivariate analysis model for predicting grade B PPAP. For the reasons mentioned above, fistula risk scores were excluded from multivariate analysis. As shown in Table 5, soft pancreatic texture (OR 2.732, 95% CI 1.590–4.695) and CRP ≥ 180 mg/L (OR 3.444, 95% CI 1.954–6.069) were the independent predictors of grade B PPAP. For predicting the model of grade C PPAP, tobacco and alcohol use, hypertension, pancreatic duct diameter <5 mm, soft pancreatic texture, liquid intake/output volume ≤ 2000 ml, operation duration >360 min, the methods of pancreatic anastomosis, and CRP ≥ 180 mg/L were enrolled compared to the non-PPAP group. As shown in Table 6, soft pancreatic texture (OR 19.298, 95% CI 1.840–2812.980), operation duration >360 min (OR 13.832, 95% CI 1.719–910.506), and the pancreatic anastomosis by using conventional duct-to-mucosa methods (OR 10.402, 95%CI 1.409-694.367) were the independent predictors of grade C PPAP.

**Table 4:** Univariate analysis of grade C PPAP.

		PPAP		*p*-value^a^	*p*-value^b^
	No. (*n* = 136)	Grade B (*n* = 140)	Grade C (*n* = 10)		
Sex ratio (M: F)	95:41	74:66	7:3	0.004	0.992
Age (year)	62 (55–69)	62 (56–69)	64 (55–70)	1.000	0.612
BMI ≥ 25, *n* (%)	81 (59.6)	67 (47.9)	7 (70.0)	0.051	0.515
Tobacco use, *n* (%)	44 (32.4)	34 (24.3)	6 (60.0)	0.137	0.075
Alcohol use, *n* (%)	14 (10.3)	10 (7.1)	3 (30.0)	0.353	0.061
AP History, *n* (%)	10 (7.4)	13 (9.3)	0 (0)	0.561	0.374
DM, *n* (%)	20 (14.7)	15 (10.7)	3 (30.0)	0.319	0.200
Hypertension, *n* (%)	35 (25.7)	34 (24.3)	6 (60.0)	0.781	0.020
Cholelithiasis, *n* (%)	32 (23.5)	38 (27.1)	1 (10.0)	0.490	0.324
Preoperative jaundice state, *n* (%)	88 (64.7)	73 (52.1)	8 (80.0)	0.034	0.325
Previous abdominal surgery, *n* (%)	30 (22.1)	30 (21.4)	2 (20.0)	0.899	0.879
ASA, *n* (%)				0.306	0.198
1	1 (0.7)	4 (2.9)	0 (0)		
2	102 (75.0)	97 (69.3)	5 (50.0)		
≥3	33 (24.3)	39 (27.9)	5 (50.0)		
Vascular resection, *n* (%)	5 (11.0)	17 (12.1)	1 (10.0)	0.773	0.920
Pancreatic duct diameter <5 mm, *n* (%)	74 (54.4)	96 (68.6)	9 (90.0)	0.016	0.028
Pancreatic texture, *n* (%)				<0.001	<0.001
Soft	46 (33.8)	86 (61.4)	10 (100.0)		
Hard	90 (66.2)	54 (38.6)	0 (0)		
Pancreatic stent, *n* (%)				0.508	0.262
Unused	14 (10.3)	9 (6.4)	0 (0)		
Internal	89 (65.4)	96 (68.6)	9 (90.0)		
External	33 (24.3)	35 (25.0)	1 (10.0)		
Bleeding ≥ 400 ml, *n* (%)	80 (58.8)	73 (52.1)	4 (40.0)	0.264	0.245
liquid intake/output volume ≤ 2000 ml, *n* (%)	34 (25.0)	34 (24.3)	5 (50.0)	0.890	0.085
Operation duration > 360 min, *n* (%)	64 (47.1)	65 (46.4)	9 (90.0)	0.916	0.009
Fistula risk scores, *n* (%)				0.044	0.066
Negligible	17 (12.5)	13 (9.3)	0 (0)		
Low	50 (36.8)	33 (23.6)	1 (10.0)		
Moderate	63 (46.3)	88 (62.9)	9 (90.0)		
High	6 (4.4)	6 (4.3)	0 (0)		
Pathology type, *n* (%)				0.395	0.153
PDAC and CP	50 (36.8)	44 (31.4)	1 (30.0)		
Other malignant	75 (55.1)	81 (57.9%)	2(58.7)		
Benign and low malignant	11 (8.1)	15 (10.7)	7 (11.3)		
Pancreatic anastomosis, *n* (%)				0.001	0.013
Modified Blumgart	85 (62.5)	60 (42.9)	3 (30.0)		
Conventional duct to mucosa	36 (26.5)	68 (48.6)	7 (70.0)		
Invagination	15 (11.0)	12 (8.6)	0 (0)		
CRP ≥ 180 mg/L, *n* (%)	30 (22.1)	70 (50.0)	7 (70.0)	<0.001	0.001

^a^ *Grade B PPAP group compared to the non-PPAP group.*

^b^ *Grade C PPAP group compared to the non-PPAP group; PPAP, postpancreatectomy acute pancreatitis; AP, acute pancreatitis; DM, diabetes mellitus; ASA, American Society of Anesthesiologists; PDAC, pancreatic duct adenocarcinoma; CP, chronic pancreatitis.*

**Table 5:** Multivariate analysis of grade B PPAP.

	OR	Multivariate	
		95% CI	*p*-value
Sex ratio (M: F)	1.577	0.879–2.827	0.126
BMI ≥ 25	0.706	0.400–1.247	0.231
Preoperative jaundice state	0.739	0.428–1.276	0.278
Pancreatic duct diameter <5 mm	1.461	0.840–2.540	0.179
Pancreatic texture (soft)	2.732	1.590–4.695	<0.001
Pancreatic anastomosis			
Modified Blumgart	0.759	0.302–1.908	0.558
Conventional duct to mucosa	1.739	0.669–4.505	0.256
Invagination	1		
CRP ≥ 180 mg/L	3.444	1.954–6.069	<0.001

**Table 6:** Multivariate analysis of grade C PPAP.

		Multivariate	
	OR	95% CI	*p*-value
Tobacco use	1.394	0.008–11.554	0.826
Alcohol use	5.534	0.248–5630.762	0.288
Hypertension	3.511	0.430–34.883	0.228
Pancreatic duct diameter <5 mm	3.987	0.376–545.662	0.269
Pancreatic texture (soft)	19.298	1.840–2812.980	0.010
liquid intake/output volume ≤ 2000 ml	1.650	0.222–14.454	0.613
Operation duration > 360 min	13.832	1.719–910.506	0.011
Pancreatic anastomosis			
Modified Blumgart	0.317	0.001–8.372	0.533
Conventional duct to mucosa	10.402	1.409–694.367	0.020
Invagination	1		
CRP ≥ 180 mg/L	3.004	0.384–26.977	0.271

### Influence of PPAP and PF on Postoperative Complications

To explore the influence of PPAP and PF on postoperative complications, the patients was divided into none of PPAP and PF occurred group, only PPAP occurred group, only PF group, and both PPAP and PF occurred group. The postoperative complications among different groups are given in Table 4. It shows that the occurrence of PPAP was independent of PF by observing 100 patients with PPAP but was not complicated with PF. Meanwhile, 50 patients suffered from both PPAP and PF.

Two questions of concern to us were statistically analyzed. First, what were the clinical consequences for the patients with PPAP compared to the patients with neither PPAP nor PF? Shown in Table 7, PPAP occurrence just significantly increased the incidence of abdominal fluid collection (*p* < 0.001); however, other severe complications such as PPH were not significantly increased. What is more, the only PPAP occurred group had longer postoperative hospital stay and spent more money on hospitalization but did not show significant difference at the level of *p* value <0.05. Second, what were clinical impacts of PPAP complicated with PF compared to PPAP alone? PPAP complicated with PF significantly increased the mortality and incidence of Clavien–Dindo ≥ IIIb complications, PPH, DGE, intraabdominal infection, abdominal fluid collection, percutaneous drainage, and unplanned secondary operation compared to the only PPAP occurred group. In addition, PPAP complicated with PF significantly increased the length of ICU and hospital stay and hospital expenses.

**Table 7:** Postoperative complications grouped by PPAP and PF occurrence.

	PPAP and PF occurrence				*p*-value	
	None (*n* = 119)	Only PPAP (*n* = 100)	Only PF (*n* = 17)	Both (*n* = 50)	*P* ^1^	*P* ^2^
Clavien-Dindo ≥ IIIb, *n* (%)	2 (1.7)	3 (3.0)	1 (5.9)	10 (20.0)	0.844	0.001
PPH, *n* (%)	11 (9.2)	13 (13.0)	5 (29.4)	18 (36.0)	0.452	0.001
A	7 (63.6)	7 (53.8)	2 (40.0)	4 (22.2)		
B	1 (9.1)	4 (30.8)	1 (20.0)	4 (22.2)		
C	3 (27.3)	2 (15.4)	2 (40.0)	10 (55.6)		
DGE, *n* (%)	47 (39.5)	36 (36.0)	13 (76.5)	35 (70.0)	0.359	<0.001
A	31 (66.0)	17 (47.2)	3 (23.1)	11 (31.4)		
B	14 (29.8)	17 (47.2)	6 (46.2)	15 (42.9)		
C	2 (4.2)	2 (5.6)	4 (30.7)	9 (25.7)		
BL, *n* (%)	1 (0.8)	0	2 (11.8)	1 (2.0)	1	0.333
Intra-abdominal infection, *n* (%)	36 (30.3)	33 (33.0)	16 (94.1)	45 (90.0)	0.663	<0.001
AKI, *n* (%)	1 (0.8)	1 (1.0)	1 (5.9)	1 (2.0)	1	1
Bowel obstruction, *n* (%)	4 (3.4)	3 (3.0)	0 (0)	1 (2.0)	1	1
Wound infection, *n* (%)	1 (0.8)	0 (0)	0 (0)	2 (4.0)	1	0.110
Abdominal fluid collection, *n* (%)	19 (16.0)	38 (38.0)	9 (52.9)	33 (66.0)	<0.001	0.001
Puncture and drainage treatment, *n* (%)	4 (3.4)	7 (7.0)	5 (29.4)	21 (42.0)	0.219	<0.001
Secondary operation, *n* (%)	3 (2.5)	3 (3.0)	1 (5.9)	9 (18.0)	1	0.004
Mortality, *n* (%)	0 (0)	1 (1.0)	0 (0)	5 (10.0)	0.457	0.027
ICU stay (day)	1.0 (0–2.0)	1.0 (0–2.0)	2.0 (1.0–5.0)	2.0 (0–3.0)	0.874	0.007
Total hospital stay (day)	22.0(18.0–27.0)	22.5(19.0–28.0)	42.0(23.5–54.0)	29.0(23.8–37.0)	0.401	<0.001
Postoperative hospital stays (day)	15.0(12.0–18.0)	16.0(14.0–18.8)	27.0(16.5–39.0)	20.5(15.8–30.5)	0.054	<0.001
Cost ($)	13,685(11,250–16,491)	14,678(12,145–170,723)	23,299(15,373–31,391)	17,758(15,497–23,908)	0.055	<0.001

*p^1^, only PPAP group compared to the none of PPAP and PF occurred group; p^2^: both PPAP and PF occurred group compared with the only PPAP group; PPAP, postpancreatectomy acute pancreatitis; PF, clinical relevant pancreatic fistula; PPH, postpancreatectomy hemorrhage; DGE, delayed gastric empty; BL, bile leakage; AKI, acute kidney injury; ICU, intensive care unit.*

## Discussion

### Incidence of PPAP

Postoperative pancreatitis after PD was brought into our attention after a patient died inevitably from severe pancreatitis in remnant pancreas in January 2016; then, we started to do targeted inspections (serum enzymology and CT examination) on suspected patients to provide an actual aid for patients' management. Therefore, the patients' cohort in this retrospective study had relatively complete data of serum enzymology and abdominal CT images. We retrieved the PubMed database; acute pancreatitis after partial pancreatectomy was first reported in 1952 (23). Recent literature started to focus on PPAP, and the occurrence of PPAP was an independent predictor of PF (5). However, there was considerable heterogeneity in previous studies due to the lack of uniform diagnostic criteria and grading system. Recently, ISGPS published the definition of PPAP (11), which was a milestone in the research of PPAP.

The incidence of PPAP varied greatly between current and past studies. According to Kriger et al. (24), the incidence of PPAP was 58.9% (178/302) by the Atlanta classification and definitions (12). Based on Connor's definition, Nahm et al. (7) found the incidence of PPAP was 62% (38/61). Most recently, Bassi et al. (5) reviewed 292 PD patients, and the incidence was 55.8% (163/292) in 2018. In the same year, the German team (6) reported that the incidence was 53% (100/190). Here, we practiced this definition published by ISGPS, and the incidence of PPAP was 52.4% in this study, which was at the same level as in current studies. However, past literature works reported the incidence of PPAP at a very low level of about 2%–3% (25, 26). Possibly due to a lack of standardized definitions in past, PPAP was diagnosed only in the condition of typical or severe clinical symptoms or life-threatening complications caused by pancreatitis. In other words, postpancreatectomy pancreatitis mentioned in the past literature probably meant the grade C PPAP (11). In this study, the incidence of grade C PPAP was 3.5%, which was comparable to the past literature (25, 26). This result provided a possible explanation for the polarization of PPAP incidence in the literature. Recent study (27) showed that necrotizing pancreatitis of the remnant pancreas confirmed by histological section was found in 33 out of 79 (41%) patients who underwent completion pancreatectomy after initial PD due to unfavorable complications. The postoperative pancreatitis not only occurred in the PD or other partial pancreatectomy but also was reported in scoliosis surgery (28), aortic dissection (29), renal transplantation (30), and gynecologic and obstetric surgery (31), and the incidence ranged from 0.29% to 5.9%.

### Diagnostic Parameters of PPAP

The diagnostic criteria of PPAP consisted of three parameters: biochemical, radiological, and clinical evidence. The elevated serum amylase greater than the upper limit showed the same diagnostic efficacy as the elevation of three times (32). Early and sustained elevation of serum amylase was thought to be more associated with postoperative complications than its peak value detected (33). The available literature (4) agreed that PPAP occurred in the early phase of postoperative period (POD 0–3). Thus, it is distinguished from the time of PF occurrence, which was defined at the later phase (POD ≥ 3) (17). Abdominal pain is an essential criterion in usual acute pancreatitis. However, abdominal pain is not a reliable diagnostic criterion after partial pancreatectomy because it could be concealed by postoperative analgesia to varying degrees (11). Early postoperative CT scans were helpful to evaluate the recovery in the surgical field. The remnant pancreas (pancreatic body and tail) was not conventionally dissected during PD procedure; however, the signs of pancreatic exudation or parenchymal changes in postoperative CT scans suggest the formation of PPAP (34). Palumbo et al. (35) suggested that the routinely postoperative CT scan after laparoscopic sleeve gastrectomy was helpful to early stratification of leakage risk. Contrast-enhanced CT in our retrospective study was less adopted except when necessary, mainly because it usually has a long waiting time for examination and might put extra burden on the kidneys, which was not suitable for patients in early postoperative phase.

### Clinical Significance of PPAP

PPAP was significantly associated with unfavorable outcomes in our study. PPAP and PF are reciprocal causation, and they can also occur independently. In this study, PF complicated with PPAP was found in 50 (74.6%) out of 67 PF patients. On the one hand, PPAP could cause cellular injury by releasing active zymogens and stimulate inflammatory response in the pancreatic parenchyma (36). In the setting of pancreatojejunostomy, zymogens can be activated by digestive juice in reconstructed digestive tract, which causes autodigestive injury in anastomotic tissue. PPAP could probably prolong the healing time of anastomosis and provide a pre-condition for the occurrence of pancreatic leakage, which then leads to PF (11). On the other hand, activated pancreatic juice leaking from dehiscence of the anastomosis pervades the remnant pancreas, which causes inflammatory damage (10). Besides, we found that 66.7% (100/150) PPAP, which was not complicated with PF, did not lead to serious complications; however, PF complicated with PPAP could cause serious complications and increase the mortality rate (10.0%). Rudis et al. found that grade C PF complicated with PPAP was observed in 4 out of 160 patients, and none of these patients survived (37). We also noticed that a large proportion of PPAP with unfavorable outcomes was complicated with PF, and PF appeared to be the major factor on outcome. Only the occurrence of PPAP may not lead to serious clinical impacts, unless grade C PPAP, which could be the cause of persistent organ failure and other severe complications.

### Risk Factors for PPAP

In this study, we found that soft pancreatic texture and CRP ≥ 180 mg/L were the independent predictors of grade B PPAP. In addition, soft pancreatic texture, operation duration >360 min, and pancreatic anastomosis by using conventional duct-to-mucosa methods were the independent risk factors for grade C PPAP. Due to the small sample size in our study, some variables occurred complete separation and quasi-complete separation. To improve the stability of the prediction model, we used Firth logistic regression to perform multivariate analysis. A retrospective study from the University of Heidelberg (6) reported a comparable incidence of PPAP and the association with CRP to our study. Notably, the pancreatic texture was observed in all patients with grade C PPAP. However, the soft texture of pancreas is a subjective index. Nahm et al. (7) reported that the acinar cell density at the pancreatic resection margin can better describe the residual pancreas than “texture,” and the density of acinar was significantly associated with PPAP. Univariate analysis showed that PPAP was more likely to take place in females, and female is also one of the independent risk factors for PEP (38). Women have a higher percentage of body fat than men, which makes the pancreas softer; hpwever, gender did not show statistical difference in multivariate analysis in this study. Bassi et al. (5) found that independent risk factors for PPAP included preoperative exocrine insufficiency, neoadjuvant therapy, additional resection of the pancreatic stump margin, soft pancreatic texture, and main pancreatic duct diameter ≤3 mm. From our retrospective data, details of extended pancreatic stump resection were not routinely recorded, and in 92 patients with pancreatic ductal adenocarcinoma, only 5 (5.4%) patients with borderline resectable pancreatic cancer received neoadjuvant chemotherapy and (or) radiotherapy, so these variables were not analyzed. Intraoperative pancreatic ischemia was thought to be a mechanism for pancreatitis (39, 40); nevertheless, this study cannot produce effective comparisons between the two groups. For the average liquid intake/output volume on the surgery day, our patients’ cohort was far beyond the “near-zero fluid” (5) (PPAP group: 2729 ml, non-PPAP group: 2721 ml).

### Managements of PPAP

Currently, the methods to prevent PPAP were very limited due to the lack of RCTs and prospective studies. The usage of pancreatic enzyme inhibitors, including octreotide and somatostatin, was not a protective factor for PPAP occurrence in this study. This was in accordance with the results of PF study (41–43). Somatostatin drugs can reduce splanchnic blood flow (44), which may increase the occurrence of pancreatitis and PF. One RCT study (45) demonstrated that prophylactic administration of ulinastatin can reduce the incidence of PPAP and also reduce the levels of amylase in serum and drain. Hydrocortisone (46) and rectal indomethacin (47) had proved that they can reduce the incidence of postoperative complications; however, these factors were not enrolled in this study due to very small sample size (*n* = 2).

The deficiency of this study was its retrospective design. Moreover, the patients were not stratified by preoperative features and surgical techniques to avoid small sample sizes, which may reduce the statistical efficiency and make clinical significance unstable. The data of preoperative amylase were lacking in this study, and the delta could better describe the inflammatory changes in the remnant pancreas than the specified threshold (48). The study is focused on the early postoperative period; however, and the impacts of PPAP on the long-term, such as recurrent pancreatitis, chronic pancreatitis, diabetes, fatty liver, and survival, were not calculated.

In conclusion, based on the structured definition and grading system of PPAP published by ISGPS, we found the incidence of PPAP after PD was at a high level (52.4%), which was in accordance with current research. Stratified by the grade of PPAP, soft pancreatic texture and CRP ≥ 180 mg/L were the independent predictors of grade B PPAP, and soft pancreatic texture, operation duration >360 min, and the pancreatic anastomosis by using conventional duct-to-mucosa methods were the independent predictors of grade C PPAP. PPAP had certain clinical practical significance on the clinical outcomes, especially when it was complicated with PF. Higher-volume multicenter and prospective studies are needed to promote a better understanding of PPAP.

## Data Availability

The raw data supporting the conclusions of this article will be made available by the authors, without undue reservation.
