# Supplementary material for: Risk Factors and Clinical Impacts of Post-Pancreatectomy Acute Pancreatitis After Pancreaticoduodenectomy: A Single-Center Retrospective Analysis of 298 Patients Based on the ISGPS Definition and Grading System
Source: Front Surg. 2022 Jul 4;9:916486. doi: 10.3389/fsurg.2022.916486 (PMC9289243; doi:10.3389/fsurg.2022.916486)
Supplement: Supplementary file 1 [file Table_1.docx]

Supplementary Table 1 Serum amylase level on POD 1-3 grouped by PPAP occurrence

|  | Serum amylase level (U/L) | |  |  |
| --- | --- | --- | --- | --- |
| POD (days) | No PPAP | PPAP | *t* | p-value |
| 1 | 159.6 ± 38.9 | 1246.6 ± 654.3 | 1.997 | 0.058 |
| 2 | 61.0 ± 6.6 | 190.9 ± 40.2 | 2.659 | 0.014 |
| 3 | 53.6 ± 14.6 | 167.8 ± 24.7 | 3.982 | 0.001 |

PPAP, post-pancreatectomy acute pancreatitis; POD, post-operative days.
